# The Effect of Mindset and Breathing Exercises on Physical and Mental Health in Persons with Spinal Cord Injury—A Pilot Feasibility Study

**DOI:** 10.3390/ijerph20186784

**Published:** 2023-09-20

**Authors:** Sonja de Groot, Frank W. L. Ettema, Christel M. C. van Leeuwen, Wendy J. Achterberg, Thomas W. J. Janssen, Sven P. Hoekstra

**Affiliations:** 1 Amsterdam Rehabilitation Research Center, Reade, 1054 HW Amsterdam, The Netherlands; t.w.j2.janssen@vu.nl; 2 Department of Human Movement Sciences, Faculty of Behavioural and Movement Sciences, Vrije Universiteit Amsterdam, Amsterdam Movement Sciences, 1081 BT Amsterdam, The Netherlands; 3 Reade Center for Rehabilitation & Rheumatology, 1054 HW Amsterdam, The Netherlands; f.ettema@reade.nl (F.W.L.E.); w.achterberg@reade.nl (W.J.A.); 4 Centre of Excellence for Rehabilitation Medicine, University Medical Centre Utrecht Brain Centre, University Medical Centre Utrecht, 3583 TM Utrecht, The Netherlands; c.v.leeuwen@dehoogstraat.nl; 5 Department of Spinal Cord Injury and Orthopedics, De Hoogstraat Rehabilitation, 3583 TM Utrecht, The Netherlands; 6 Department of Rehabilitation Medicine, University of Texas Health Science Center at San Antonio, San Antonio, TX 78229-3900, USA; s.p.hoekstra@lboro.ac.uk; 7 School of Sport, Exercise and Health Sciences, Loughborough University, Loughborough LE11 3TU, UK

**Keywords:** preventative medicine, Wim Hof Method, secondary complications, spirometry, spinal cord injury

## Abstract

This study investigated the feasibility and efficacy of mindset and breathing exercises (Wim Hof Method (WHM)) on physical and mental health in persons with spinal cord injury (SCI). Ten individuals with SCI participated in this pilot study. These ten participants followed a 4-week WHM intervention, with one weekly group session in the rehabilitation center and daily practice at home using the WHM app. An in-person exit-interview was conducted post-intervention to collect qualitative information on participants’ experiences, regarding the feasibility and effects of the intervention. Furthermore, tests and questionnaires were administered pre- and post-intervention to assess physical and mental health outcomes. Adherence to the weekly in-person meetings was excellent and no adverse events occurred. Physical and mental health outcomes in this small sample size showed some pre–post differences. This pilot feasibility study provides preliminary evidence supporting the feasibility and efficacy of the WHM, including mindset and breathing exercises, on physical and mental health of people with SCI. These results warrant a randomized-controlled trial, including cold exposure, of this novel intervention in people with SCI.

## 1. Introduction

Neurological disorders, such as a spinal cord injury (SCI), are serious medical conditions that cause functional, psychological and socioeconomic disorder. Long-term secondary medical complications are common and play an important role in the continuum of care [1]. Complications are a frequent cause of morbidity and mortality and lead to increased rates of rehospitalization, loss of employability and decreased quality of life [1].

Respiratory dysfunction can be one of the complications of a high-level SCI [2], including insufficiency of respiratory muscles, reduction in vital capacity and an ineffective cough [3]. Respiratory complications associated with SCI occur in both acute and chronic stages [2]. A SCI also has mental consequences. For example, the perceived stress score is higher in persons with SCI than that found in the general population [4]. In the same cohort, stress was directly related to depressive symptomatology and anxiety, and inversely to life satisfaction [4]. Furthermore, chronic pain is one of the frequent secondary complications, with up to 80% of patients with SCI reporting to suffer from it [5]. Chronic pain may lead to functional disability and emotional discomfort and may impact negatively on community participation and quality of life [6]. Another common secondary impairment after SCI is spasticity, which affects 70% of patients with SCI and causes considerable disability and discomfort for many [7]. Considering the multiple co-morbidities related to SCI, rehabilitation strategies are multidisciplinary and focus on working with the patient physically and psychologically to maximize their neurological recovery and general health but also to prevent or manage the above-mentioned secondary complications. 

An intervention that has shown several positive effects in different populations is the Wim Hof Method (WHM). The WHM is based on three elements: (1) breathing exercises, (2) mindset, i.e., commitment to focus on the task without being distracted, and (3) gradual cold exposure (cold showers, or cold water immersion) [8]. A few studies have investigated the effects of the WHM in different populations and showed promising findings on physiological and psychological stress responses. Practicing the WHM techniques induced intermittent respiratory alkalosis and hypoxia, as well as a profound increase in plasma epinephrine concentration in healthy individuals [9]. These changes correlated with favorable changes in resting inflammatory markers [9,10]. Additionally, in patients with active axial spondylarthritis, positive effects of the WHM were found on the inflammatory profile as well as on quality of life in the intervention compared to the control group [8]. Reduction in depressive symptoms, also when the WHM was prescribed entirely remotely, was found in two other studies [11,12]. It would be interesting to study whether the WHM also induces positive effects in people with an SCI, who have a different pathophysiology compared to the healthy individuals and patients that were investigated in the studies mentioned above. 

In people with SCI, the breathing exercises of the WHM, with deep inhalations, may improve respiratory function and subsequently reduce respiratory complications [13]. Daily deep breathing exercises can result in favorable physiological adaptations, including reduced blood pressure and sympathetic activity, and can reduce stress [14]. Mindset exercises, i.e., focusing on the task without being distracted and using willpower, self-control and commitment to perform the WHM daily, are also part of mindfulness, which has been shown to decrease perceived stress in people with multiple sclerosis [15]. Stress reduction, following breathing and mindset exercises could also have an effect on spasticity and pain in people with SCI [16]. Lastly, voluntary exposure to cold water may have beneficial health effects on, among other things, the immune system, pain, and mental health [17]. However, it is important to be able to control breathing before being exposed to cold water and it is not known whether persons with SCI are able to do so. 

Although the WHM might induce positive effects in people with SCI, the feasibility and efficacy of the WHM on physical and mental health have not yet been tested in this population. Therefore, this pilot study aimed to explore the feasibility and potential effects of part of the WHM method (breathing exercises and mindset) on physical and mental health in persons with SCI. The focus was on these two parts of the WHM method since breathing and mindset exercises are prerequisites for cold exposure and were anticipated to be easy and safe to implement in clinical practice and at home. 

## 2. Materials and Methods

### 2.1. Participants

Ten individuals with SCI (four males), a convenience sample of outpatients of a single rehabilitation center, participated in the WHM pilot (Table 1). Inclusion criteria were an age between 18–75 years and a time since injury longer than 1 year. These individuals were selected with the goal of having a variety of ages, time since injuries and SCI levels and completeness in the sample. Their median age was 51 (Interquartile range (IQR): 38.0–60.0) years and their median time since injury was 3.5 (2.5–17.0) years.

### 2.2. Design

A single arm pre–post test pilot study was performed. The participants engaged in a 4-week WHM intervention focusing on breathing exercises and mindset, with one weekly practice session at the rehabilitation center, led by a WHM certified instructor. Besides these weekly sessions, the participants were requested to practice daily at home with the WHM app (Wim Hof Method Breathing and Cold mobile app, WHM services; available in the Google Play and Apple store).

### 2.3. Intervention

Breathing exercises: First, participants were asked to breathe deeply for an average of 30 breaths, i.e., deep inhalations and relaxed expirations. Subsequently, the participants exhaled and held their breath in an unforced manner until they felt a stimulus to inhale (“retention phase”). The duration of breath retention is at the discretion of the participants themselves. For safety, they were instructed not to hold their breath longer than 3.5 min. Breath retention was followed by a deep inhalation breath, held for 15 s. Subsequently, a new cycle of deep breathing began. In total, three cycles were performed. 

Mindset: The mindset was trained during the breathing exercises. Commitment to focusing on the task, i.e., deeply breathing and holding their breath, without being distracted by other thoughts, was encouraged during the group sessions, and required during the home-based sessions. Willpower, self-control, and commitment are considered important parts of the WHM.

The daily breathing exercises, including the mindset, took about 30 min.

### 2.4. Feasibility

Adherence of the one-weekly practice sessions at the rehabilitation center was tracked. Furthermore, the data completion rate of the tests and questionnaires before and after the intervention was assessed. Lastly, adverse events were monitored during the study.

At the end of the intervention an in-person exit-interview was conducted to collect qualitative information on participants’ experiences, regarding the feasibility and effects of the intervention. 

### 2.5. Efficacy—Physical Health

The following tests and questionnaires were administered at T1 (before the intervention) and T2 (in the week after the intervention period). 

Respiratory function was assessed according to a standardized protocol [18] with the EasyOne Air (NDD Medical Technologies, Andover, MA, USA). The following parameters were measured: forced vital capacity (FVC, liter), forced expiratory volume in 1 s (FEV1, liter), and peak expiratory flow (PEF, liter/s). The FVC and FEV1 were also expressed as a percentage of the predicted values based on able-bodied persons of the same age, sex, and height. Participants had to breathe through a mouthpiece while wearing a nose clip. Each measurement was performed until three reproducible measurements within at most ±5% difference were registered. The highest measured value of each parameter was used for further analysis.

Blood pressure (diastolic and systolic) was assessed once through an automated arterial pressure monitor while seated. 

Chronic pain was assessed with the Dutch translation of the international SCI pain basic data set [19]. The questions ‘Average pain intensity in the last week’ and ‘In general, how much has pain interfered with your day-to-day activities in the last week’ were scored on a numerical rating scale (ranging from 0 = “No pain” or “No interference” to a maximum of 10 = “Pain as bad as you can imagine” or “Extreme interference”). These questions were filled out for nociceptive pain, neuropathic pain, and other pain separately. 

Spasticity was measured by two questions addressing the experienced degree and discomfort of spasticity. The answers ranged from 0 (no spasticity) to 10 (maximal spasticity). The hindrance of spasticity perceived by the participants was also assessed with a questionnaire [20]. The hindrance participants perceived due to spasticity was assessed for different aspects of daily living activities: sleeping, making transfers, washing and clothing, wheelchair maneuvering and propulsion, and “others” [20]. A sumscore was calculated, which ranged from 0 (no hindrance) to 10 (a lot of hindrance during all activities) [20].

### 2.6. Efficacy—Mental Health

Hyperventilation was assessed with the Nijmegen Hyperventilation Syndrome Questionnaire [21]. It consists of 16 items related to symptoms of hyperventilation scored on a 5-point Likert scale ranging from ‘never’ to ‘very often’. A sumscore of more than 23 suggests significant hyperventilation symptoms. This questionnaire mainly reflects the subjective, psychological dimension of breathing and its response to stress. 

Mental health was assessed through the Mental Health Index-5 (MHI-5), a subscale of the Dutch translation of the 36-Item Short Form Health Survey (SF-36) [22]. The MHI-5 consists of five items, pertaining to nervousness, sadness, peacefulness, mood and happiness. Respondents rate the frequency of each item during the previous 4 weeks on a 6-point Likert-response scale (1 = all of the time, 2 = most of the time, 3 = a good bit of the time, 4 = some of the time, 5 = a little of the time, 6 = none of the time). A total score was computed by summing and transforming the five-item scores into a score between 0 (lowest mental health) and 100 (highest mental health). A score of ≤72 points refers to mental health problems, and of ≤60 points refers to severe mental health problems [23].

Fatigue was measured using the Fatigue Severity Scale (FSS), a validated questionnaire assessing the perceived impact of fatigue on an individual’s daily functioning. The range of answer possibilities is 1–7, and the total FSS score is the mean of nine questions. The mean Dutch general population score is 2.9 ± 1.1 [24]. ‘Severe fatigue’ was defined as a score on the FSS of >2 SD above the mean score in the general population (FSS ≥ 5.1) [24]. ‘Fatigue’ was defined as a score on the FSS of >1 SD above the mean score in healthy individuals (FSS > 4.0) [24]. 

Sleep quality was assessed with the Pittsburgh Sleep Quality Index, a 9-item questionnaire [25]. The items target sleep habits and perceived sleep quality over the past month. Responses to the individual items are converted to a score from 0 to 3, from which a total sum is calculated.

### 2.7. Statistics

SPSS Statistics 26 was used for all statistical analyses. Non-parametric tests were performed since not all outcome measures were normally distributed and the sample size of this pilot study was small. Therefore, a Wilcoxon signed rank test was performed to check for differences in outcome measures between the pre- and post-tests. The effect size r was calculated based on the Z-value, a statistical measurement that describes a value’s relationship to the mean of a group of values, and the sample size (N) (r = Z/√N) and referred to as small (r = 0.10), medium (r = 0.30) and large (r = 0.50). Alpha was set at *p* = 0.05. Furthermore, scatterplots were made in R (ggplot2 function) to show the individual differences between the pre- and post-tests. 

## 3. Results

### 3.1. Feasibility

All ten participants attended all four weekly practice sessions at the rehabilitation center, indicating an excellent adherence to the in-person meetings.

The data collection completion rate was overall good. Blood pressure results were missing for one person at the pre-test. The pain questionnaire was only filled out when the question was applicable to the participant for that specific pain. This led to an N ranging between 4 and 8 for the pain intensity and pain interference questions. Furthermore, some of these questions were filled out by only N = 2 at the pre- and post-test. 

No adverse events occurred during the intervention period. 

Table 2 shows the experiences of the participants regarding the WHM intervention. Participants experienced multiple positive physical and mental changes. Better sleep, feeling more relaxed and having more energy were mentioned the most (by N = 4), followed by experiencing less pain (N = 3). Lack of motivation to perform the breathing exercises was mentioned most frequently as a barrier for the WHM intervention (N = 4). This was followed by “costs a lot of time/effort”, “difficult to perform alone” (i.e., easier to perform the exercises in a group), and “difficult to give up control”, i.e., let emotions out (all N = 2).

### 3.2. Physical Health

**Figure 1:**
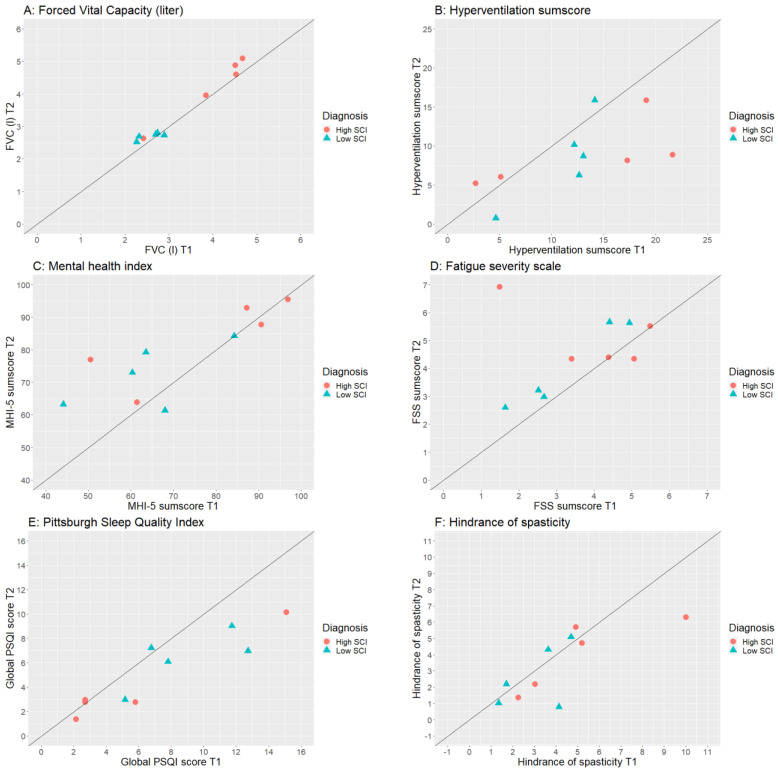
Overview of the individual data at the pre-test (T1, *x*-axis) and post-test (T2, *y*-axis) for (**A**) Forced vital capacity (FVC), (**B**) Hyperventilation sumscore, (**C**) Mental health index (MHI-5), (**D**) Fatigue severity scale (FSS), (**E**) Pittsburgh sleep quality index (PSQI), and (**F**) Hindrance of spasticity. High SCI: spinal cord injury at or above Th6; Low SCI: spinal cord injury below Th6; The diagonal line is the line of identity (loi): when the dot is on this line it indicates that the pre and post values are exactly the same. Favorable changes are seen when the dots are on the left side of the loi for the FVC, MHI-5 and on the right side of the loi for the hyperventilation sumscore, FSS, Global PSQI score, and hindrance of spasticity.

Table 3 shows the median and interquartile range (IQR) of all outcome parameters at the pre- and post-tests and whether they changed statistically significantly during the WHM intervention. Figure 1 shows the individual data of a selection of outcome measures, the plots of the other outcome measures can be found in the (Supplemental File Figure S1).

A statistically significant improvement was found in the respiratory parameter FVC from T1 to T2 (Figure 1A). The effect size was large for all five respiratory parameters. In contrast, blood pressure did not significantly change over time. 

No significant changes were found in pain intensity or pain interference. The degree of spasticity as well as the hindrance of spasticity during daily life activities did not change significantly between T1 and T2. The discomfort due to spasticity showed a trend (*p* = 0.088) towards less discomfort or hindrance (Figure 1F). Three of the participants (30%) indicated no hindrance at all or a little hindrance during only one activity before the WHM intervention, compared to five (50%) after the intervention.

### 3.3. Mental Health

At baseline, none of the participants showed significant hyperventilation symptoms, indicated by a sumscore below 23 for each individual. Nonetheless, the hyperventilation sumscore was significantly decreased following the intervention (Figure 1B). 

MHI-5 showed a trend (*p* = 0.091) for improvement between T1 and T2. Figure 1C shows that participants with a low MHI-5 score at T1 improved most. The number of participants with severe mental health problems (≤60 points) decreased from four to one, the number of people with mental health problems (>60 and ≤72 points) went from two to three, and the group with no mental health problems (>72) increased from four to six (*p* = 0.034).

The FSS showed an unfavorable and significant increase between measurements (Figure 1D). This was underscored by a shift of participants from the no fatigue (pre: N = 5 to post: N = 3) or fatigue (pre: N = 3 to post: N = 3) categories to the severe fatigue category (pre: N = 2 to post: N = 4).

Sleep quality showed a significant improvement (*p* = 0.018) (Figure 1E).

## 4. Discussion

The present pilot feasibility study showed that the WHM, involving breathing exercises and mindset, can be safely and feasibly applied in people with SCI. Furthermore, preliminary findings showed that participants experienced multiple positive changes and also some pre–post differences were found in the outcomes of the physical tests and questionnaires. A larger scale trial, including a control group, to investigate the WHM intervention in people with SCI is, therefore, warranted.

All participants attended the four weekly practice sessions at the rehabilitation center, indicating excellent adherence. Unfortunately, we did not formally monitor the adherence of the home-based practice with the WHM app, besides discussing weekly how the home training went. Although participants received thorough instructions on the daily sessions they were asked to perform at home, these may have diverged in duration and intensity between participants. A recommendation for future studies is to closely monitor the sessions at home via, e.g., a diary, that addresses the duration and intensity of the completed sessions.

No adverse events were observed in this group, indicating that the mindset and breathing exercises can be safely performed; also at home when given good instructions. 

The physical tests, as well as the questionnaires, were administered successfully, except for the pain intensity and interference questions. Since the pain questions targeted three types of pain separately (nociceptive pain, neuropathic pain and other pain) and the questions were only filled out when the question was applicable to the participant for that specific pain, the sample sizes were quite small per pain question (N = 5–6 post intervention and sometimes N = 2 for participants who filled out that question at the pre- and post-test). It is, therefore, recommended to use another pain questionnaire in future (intervention) studies. 

Our study has some strengths and limitations. Accessibility is one of the primary strengths of the WHM. The WHM intervention can be performed totally remotely, as is further underscored by a previous study [11]. However, two of our participants indicated that it was difficult to perform the WHM alone at home. Perhaps a group-based online intervention might be effective as well. Lack of motivation to practice the WHM was also an important barrier (mentioned by N = 4) and might also be related to practicing the WHM alone at home. Additionally, the home-based nature of the intervention may have resulted in slightly altered durations and intensities of the sessions, in turn reducing internal validity. However, with group-based interventions, participants are less flexible in scheduling the practice time, which might create other barriers. Moreover, the home-based aspect of WHM removes the often-cited transportation barrier for study and leisure participation [26]. 

Since this is an exploratory study, we did not adjust for multiple hypothesis testing. It is noteworthy, however, that if we had done so, none of the outcomes would have been significantly different between the pre- and post-test. A randomized-controlled study, including a control group, is therefore necessary to formally investigate the efficacy of the WHM intervention. Furthermore, the duration of the intervention, i.e., 4 weeks, was rather short due to practical (clinical) reasons. Additionally, as is common in rehabilitation studies, the sample was heterogeneous and consisted of individuals with different SCI lesion levels, and related physical capacities. Finally, subjective outcomes reported through questionnaires may be influenced by motivational aspects associated with participating in a study investigating a novel intervention. 

This pilot study showed that the WHM intervention, including mindset and breathing exercises, was feasible and safe for people with SCI and might lead to improvements in physical and mental health outcomes. Therefore, based on this pilot study, a three-arm randomized-controlled trial (RCT) was developed (see for details: ClinicalTrials.gov ID: NCT05704322). This RCT will include two intervention groups (1) mindset and breathing exercises; (2) mindset and breathing exercises plus cold exposure, and a control group, who will only receive usual care. The outcomes will be partly similar to the current pilot study, but additional measurements will be added, i.e., blood samples will be collected to determine circulating concentrations of inflammatory markers and lipids. A previous study (focusing on the inflammatory response in healthy male volunteers) showed that cold exposure training alone did not relevantly modulate the inflammatory response, in contrast to only breathing exercises. However, cold exposure training significantly enhanced the immunomodulatory effects of the breathing exercise [10]. In that respect, it is interesting to investigate the additional effect of cold exposure in people with SCI. However, the primary outcome of the RCT will be mental health, assessed with the MHI-5. Lastly, as mentioned before, the duration of the pilot intervention, i.e., 4 weeks, was rather short and, therefore, the RCT intervention will be 7 weeks. 

## 5. Conclusions

This study provides preliminary support for the feasibility and efficacy of the WHM, including mindset and breathing exercises, to improve a range of physical and mental health outcomes in individuals with SCI. These results warrant follow-up through a randomized-controlled trial, including a usual care (control) group and a group that engages in additional cold-water exposure (i.e., the third component of the WHM) and a longer intervention duration, to test the efficacy of this novel intervention in people with SCI through an appropriately powered trial.

## Figures and Tables

**Table 1:** Patient characteristics.

Participant	Sex	Age (Years)	Spinal Cord Injury Level and Completeness	Time Since Injury (Years)
1	M	56	C3—AIS C	1
2	M	46	C3–4—AIS A	3
3	M	49	Th3—AIS B	4
4	M	41	Th6—AIS A	3
5	F	31	Th6—AIS D	1
6	F	27	Th7—AIS C	4
7	F	62	Th10—AIS A	3
8	F	53	Th10—AIS C	44
9	F	59	L2—AIS D	13
10	F	62	L3—AIS D	29

NB: M: male; F: female; C: cervical; Th: thoracal; L: lumbal; AIS: ASIA Impairment Scale.

**Table 2:** Results of the in-person exit-interview (N = 10) to collect qualitative information on participants’ experiences with the intervention.

What Has the Training Achieved?	N	What Was Difficult about the Training?	N
Better sleep	4	Lack of motivation, self-discipline	4
More relaxed	4	Cost a lot of time/effort	2
More energy	4	Difficult alone, easier in group	2
Less pain	3	Giving up control	2
Physical improvement (e.g., more strength in legs)	2	First round of breathing exercise needed to get into it	1
Better activation of the lungs	2	More difficult without music	1
Less spasms	2	Need more and longer guidance	1
Better concentration	2	I was skeptical	1
Less problems with cold body	1	I had less control over diaphragm during the training, now I have more control again	1
Can breathe through nose when lying supine	1	Focus on thoughts before/after exercises	1
Core is more active	1		
Better cough	1		
Better fear control	1		
Better mood	1		

**Table 3:** Descriptives of the different outcome measures at the pre-test (T1) and post-test (T2) and results of the Wilcoxon signed rank test (N = 10).

	T1		T2		*p*-Value	Effect Size
Outcome Measure	N	Median (IQR)	N	Median (IQR)		
Respiratory function						
FVC (l)	10	2.8 (2.4–4.5)	10	2.8 (2.7–4.7)	0.022	0.67
%FVC (%)	10	76.0 (67.0–90.0)	10	79.0 (72.0–92.0)	0.036	0.66
FEV1 (l)	10	2.3 (2.1–3.6)	10	2.4 (2.2–3.7)	0.059	0.60
%FEV1 (%)	10	84.0 (69.0–92.0)	10	85.0 (71.0–94.0)	0.102	0.52
PEF (l/s)	10	5.4 (4.4–8.8)	10	6.6 (5.3–8.6)	0.059	0.60
Blood pressure (mm Hg)						
Systolic	9	121.0 (112.0–141.0)	10	121.0 (108.0–121.0)	0.574	0.19
Diastolic	9	75.0 (64.0–90.0)	10	76.0 (68.0–90.0)	0.477	0.24
Average pain intensity last week? (0–10)						
Nociceptive pain	4	6.0 (3.3–8.0)	5	3.0 (2.5–5.0)	0.317	0.71
Neuropathic pain	7	5.0 (3.0–8.0)	6	6.5 (2.8–7.8)	0.713	0.15
Other pain	8	3.0 (2.0–6.8)	5	2.0 (2.0–4.0)	0.059	0.95
Pain interference in ADL last week? (0–10)						
Nociceptive pain	4	4.5 (0.8–7.5)	5	1.0 (0.0–2.5)	0.655	0.32
Neuropathic pain	7	3.0 (0.0–6.0)	6	0.5 (0.0–3.0)	0.197	0.53
Other pain	8	0.0 (0.0–2.8)	5	0.0 (0.0–0.0)	0.109	0.80
Spasticity						
Degree (0–10)	10	3.0 (0.0–7.8)	10	2.0 (0.8–4.3)	0.320	0.31
Discomfort (0–10)	10	3.0 (1.8–7.0)	10	2.5 (0.0–3.5)	0.088	0.54
Hindrance of spasticity	10	4.0 (2.0–5.0)	10	3.0 (1.0–5.3)	0.131	0.48
Hyperventilation	10	13.0 (5.0–17.5)	10	8.5 (5.8–11.5)	0.036	0.66
MHI-5	10	66.0 (58.0–89.0)	10	78.0 (64.0–89.0)	0.091	0.53
Fatigue severity scale	10	3.9 (2.2–4.9)	10	4.4 (3.2–5.7)	0.021	0.73
Global PSQI score	10	6.5 (3.0–12.2)	10	4.5 (3.0–7.5)	0.018	0.75

NB: N: sample size; IQR: interquartile range; FVC: forced vital capacity; FEV: forced expiratory volume, PEF: peak expiratory flow, ADL: activities of daily living, MHI: mental health index; PSQI: Pittsburgh Sleep Quality Index.

## Data Availability

The datasets generated during and/or analyzed during the current study are available from the corresponding author on reasonable request.

## Supplementary Materials

The following supporting information can be downloaded at: https://www.mdpi.com/article/10.3390/ijerph20186784/s1, Figure S1: Overview of the individual data at the pre-test (T1, x-axis) and post-test (T2, y-axis) for (A) Forced expiratory volume in 1 second (FEV1), (B) Peak expiratory flow (PEF), (C) Systolic blood pressure, (D) Diastolic blood pressure, (E) Nociceptive pain intensity, (F) Nociceptive pain interference, (G) Neuropathic pain intensity, (H) Neuropathic pain interference, (I) Other pain intensity, (J) Other pain interference, (K) Spasticity degree, and (L) Spasticity discomfort. High SCI: spinal cord injury at or above Th6; Low SCI: spinal cord injury below Th6; The diagonal line is the line of identity (loi): when the dot is on this line it indicates that the pre and post values are exactly the same.

## References

[1] McKinley W.O., Jackson A.B., Cardenas D.D., DeVivo M.J. (1999). Long-term medical complications after traumatic spinal cord injury: A regional model systems analysis. Arch. Phys. Med. Rehabil..

[2] Zimmer M.B., Nantwi K., Goshgarian H.G. (2007). Effect of spinal cord injury on the respiratory system: Basic research and current clinical treatment options. J. Spinal Cord Med..

[3] Brown R., DiMarco A.F., Hoit J.D., Garshick E. (2006). Respiratory dysfunction and management in spinal cord injury. Respir. Care.

[4] Rintala D.H., Robinson-Whelen S., Matamoros R. (2005). Subjective stress in male veterans with spinal cord injury. J. Rehabil. Res. Dev..

[5] Rekand T., Hagen E.M., Grønning M. (2012). Chronic pain following spinal cord injury. Tidsskr. Nor. Laegeforen..

[6] Middleton J.W., Leong G., Mann L. (2008). Management of spinal cord injury in general practice—Part 2. Aust. Fam. Physician.

[7] Rekand T., Hagen E.M., Grønning M. (2012). Spasticity following spinal cord injury. Tidsskr. Nor. Laegeforen..

[8] Buijze G.A., De Jong H.M.Y., Kox M., van de Sande M.G., Van Schaardenburg D., Van Vugt R.M., Popa C.D., Pickkers P., Baeten D.L.P. (2019). An add-on training program involving breathing exercises, cold exposure, and meditation attenuates inflammation and disease activity in axial spondyloarthritis—A proof of concept trial. PLoS ONE.

[9] Kox M., Stoffels M., Smeekens S.P., van Alfen N., Gomes M., Eijsvogels T.M., Hopman M.T., van der Hoeven J.G., Netea M.G., Pickkers P. (2012). The influence of concentration/meditation on autonomic nervous system activity and the innate immune response: A case study. Psychosom. Med..

[10] Zwaag J., Naaktgeboren R., van Herwaarden A.E., Pickkers P., Kox M. (2022). The Effects of Cold Exposure Training and a Breathing Exercise on the Inflammatory Response in Humans: A Pilot Study. Psychosom. Med..

[11] Faid T., Van Gordon W., Taylor E.C. (2022). Breathing Exercises, Cold-Water Immersion, and Meditation: Mind-Body Practices Lead to Reduced Stress and Enhanced Well-Being. Adv. Mind Body Med..

[12] Petraskova Touskova T., Bob P., Bares Z., Vanickova Z., Nyvlt D., Raboch J. (2022). A novel Wim Hof psychophysiological training program to reduce stress responses during an Antarctic expedition. J. Int. Med. Res..

[13] Lemos J.R., da Cunha F.A., Lopes A.J., Guimarães F.S., do Amaral Vasconcellos F.V., Dos Santos Vigário P. (2020). Respiratory muscle training in non-athletes and athletes with spinal cord injury: A systematic review of the effects on pulmonary function, respiratory muscle strength and endurance, and cardiorespiratory fitness based on the FITT principle of exercise prescription. J. Back Musculoskelet. Rehabil..

[14] Tavoian D., Craighead D.H. (2023). Deep breathing exercise at work: Potential applications and impact. Front. Physiol..

[15] Simpson R., Mair F.S., Mercer S.W. (2017). Mindfulness-based stress reduction for people with multiple sclerosis—A feasibility randomised controlled trial. BMC Neurol..

[16] Hearn J.H., Cross A. (2020). Mindfulness for pain, depression, anxiety, and quality of life in people with spinal cord injury: A systematic review. BMC Neurol..

[17] Esperland D., de Weerd L., Mercer J.B. (2022). Health effects of voluntary exposure to cold water—A continuing subject of debate. Int. J. Circumpolar Health.

[18] (1995). Standardization of Spirometry, 1994 Update. American Thoracic Society. Am. J. Respir. Crit. Care Med..

[19] Nachtegaal J., van Langeveld S.A., Slootman H., Post M.W.M. (2018). Implementation of a Standardized Dataset for Collecting Information on Patients with Spinal Cord Injury. Top. Spinal Cord Inj. Rehabil..

[20] van Cooten I.P., Snoek G.J., Nene A.V., de Groot S., Post M.W. (2015). Functional hindrance due to spasticity in individuals with spinal cord injury during inpatient rehabilitation and 1 year thereafter. Spinal Cord.

[21] van Dixhoorn J., Duivenvoorden H.J. (1985). Efficacy of Nijmegen Questionnaire in recognition of the hyperventilation syndrome. J. Psychosom. Res..

[22] van Leeuwen C.M., van der Woude L.H., Post M.W. (2012). Validity of the mental health subscale of the SF-36 in persons with spinal cord injury. Spinal Cord.

[23] Hoeymans N., Garssen A.A., Westert G.P., Verhaak P.F. (2004). Measuring mental health of the Dutch population: A comparison of the GHQ-12 and the MHI-5. Health Qual. Life Outcomes.

[24] Merkies I.S., Schmitz P.I., Samijn J.P., van der Meché F.G., van Doorn P.A. (1999). Fatigue in immune-mediated polyneuropathies. European Inflammatory Neuropathy Cause and Treatment (INCAT) Group. Neurology.

[25] Buysse D.J., Reynolds C.F., Monk T.H., Berman S.R., Kupfer D.J. (1989). The Pittsburgh Sleep Quality Index: A new instrument for psychiatric practice and research. Psychiatry Res..

[26] Anderson K.D., Cowan R.E., Horsewell J. (2016). Facilitators and Barriers to Spinal Cord Injury Clinical Trial Participation: Multi-National Perspective of People Living with Spinal Cord Injury. J. Neurotrauma.

