# Supplementary material for: The Effect of Mindset and Breathing Exercises on Physical and Mental Health in Persons with Spinal Cord Injury—A Pilot Feasibility Study
Source: Int J Environ Res Public Health. 2023 Sep 20;20(18):6784. doi: 10.3390/ijerph20186784 (PMC10531230; doi:10.3390/ijerph20186784)
Supplement: Supplementary file 1 [file ijerph-20-06784-s001.zip › ijerph-2580526-supplementary.pdf]

International Journal of
Environmental Research and Public Health

MDPI

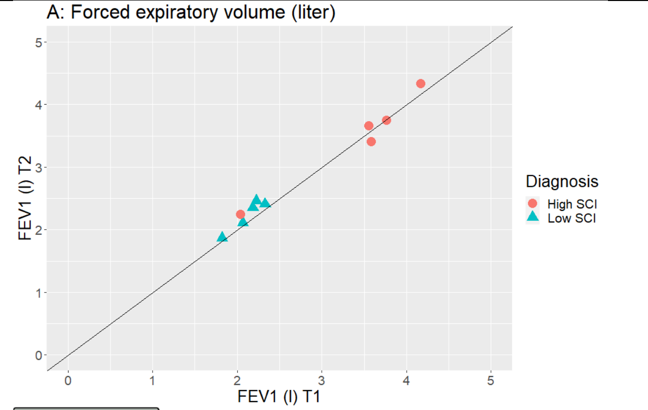

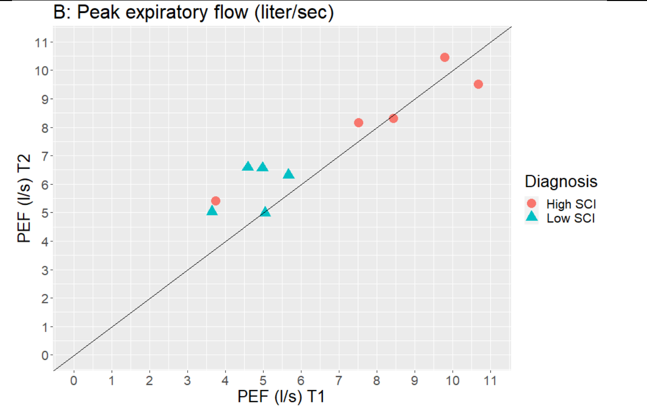

Copyright: © 2023 by the authors. Licensee MDPI, Basel, Switzerland. This article is an open access article distributed under the terms and conditions of the Creative Commons Attribution (CC BY) license (https://creativecommons.org/licenses/by/4.0/).

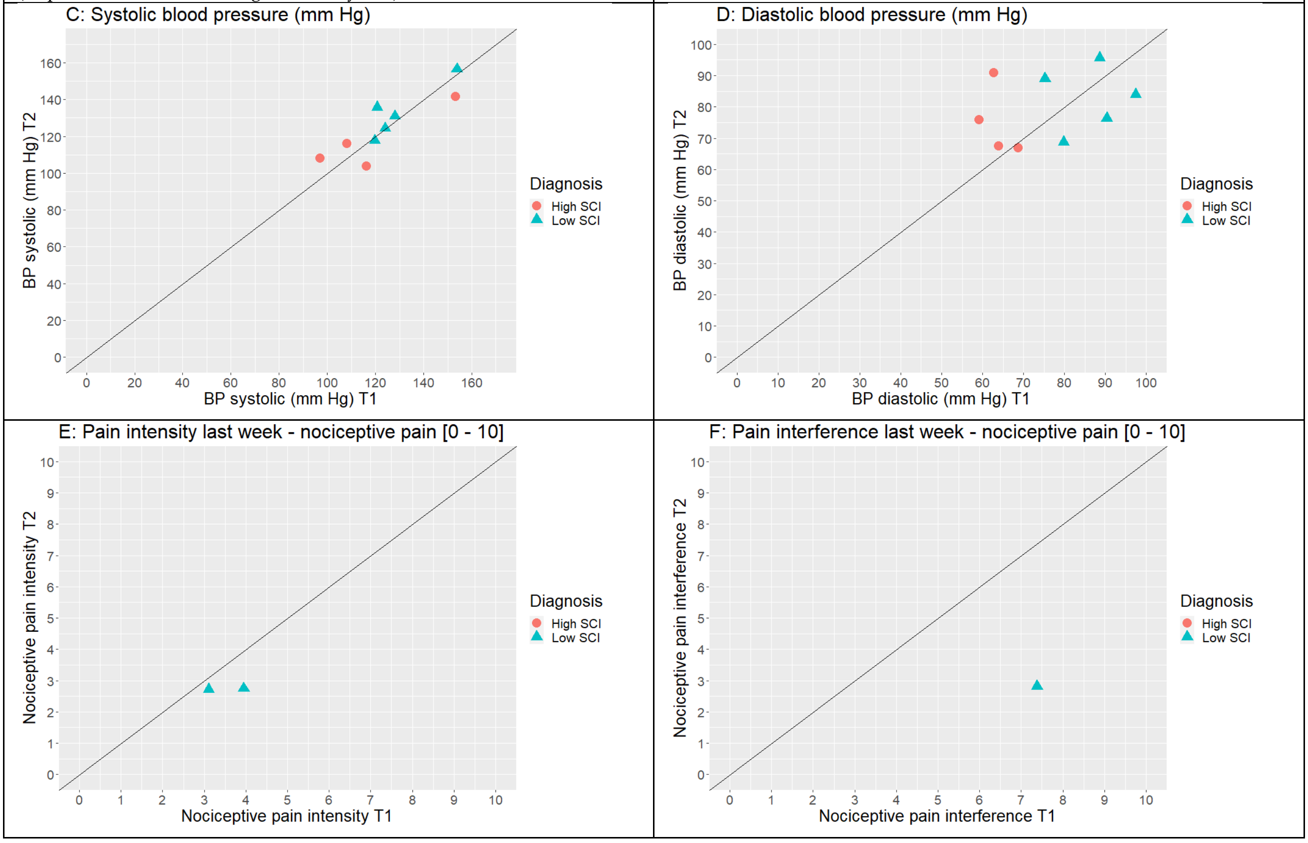

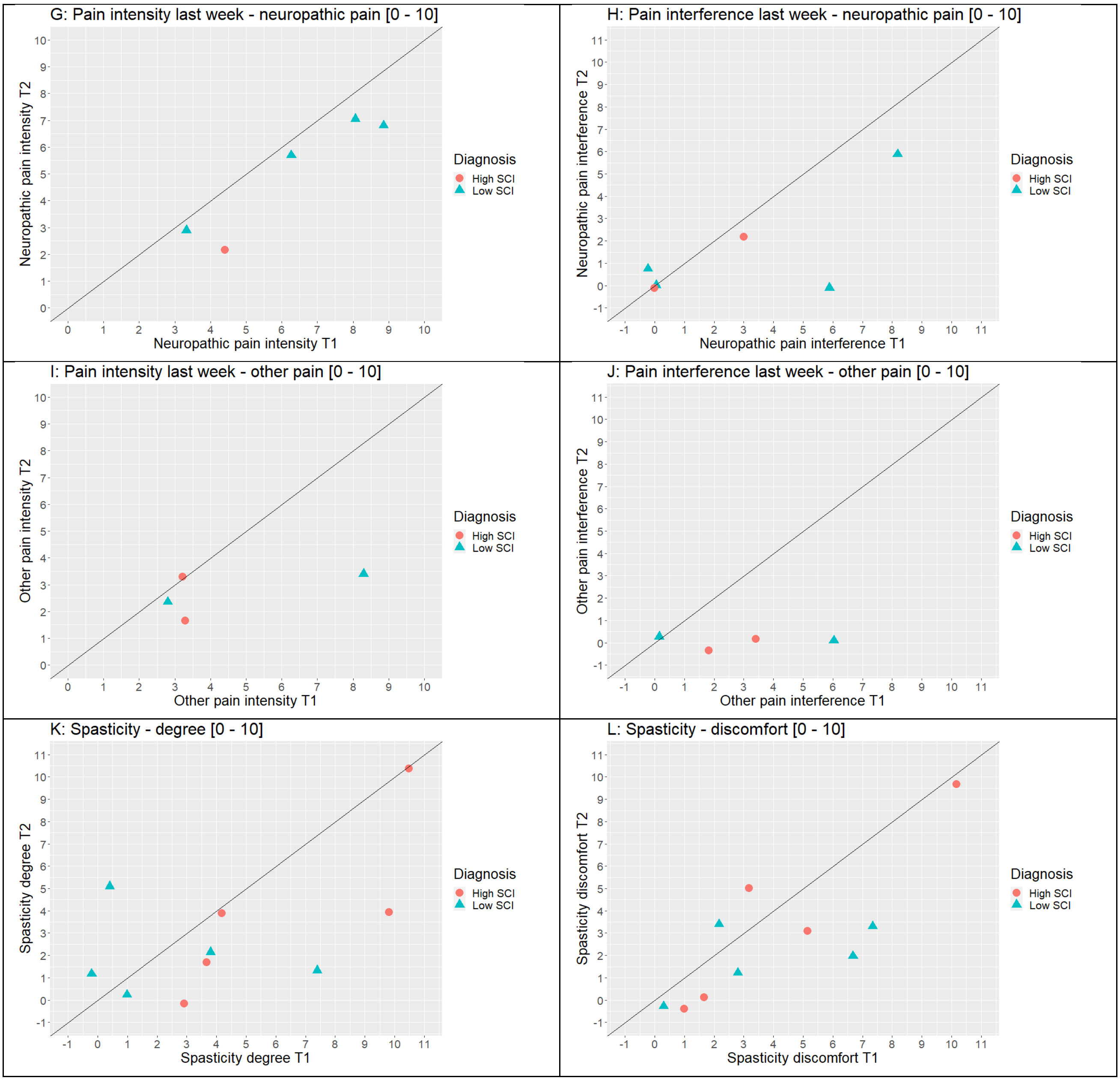

Figure S1. Overview of the individual data at the pre-test (T1, x-axis) and post-test (T2, y-axis) for A) Forced expiratory volume in 1 second (FEV1), B) Peak expiratory flow (PEF), C) Systolic blood pressure, D) Diastolic blood pressure, E) Nociceptive pain intensity, F) Nociceptive pain interference, G) Neuropathic pain intensity, H) Neuropathic pain interference, I) Other pain intensity, J) Other pain interference, K) Spasticity degree, and L) Spasticity discomfort. High SCI: spinal cord injury at or above Th6; Low SCI: spinal cord injury below Th6; The diagonal line is the line of identity (loi): when the dot is on this line it indicates that the pre and post values are exactly the same.

Disclaimer/Publisher's Note: The statements, opinions and data contained in all publications are solely those of the individual author(s) and contributor(s) and not of MDPI and/or the editor(s). MDPI and/or the editor(s) disclaim responsibility for any injury to people or property resulting from any ideas, methods, instructions or products referred to in the content.
